# Low Human Papillomavirus Vaccination in a Low-Income Urban Population

**DOI:** 10.1177/10901981231179938

**Published:** 2023-06-17

**Authors:** Elizabeth M. Rojo, Kelly D. Taylor, Willi McFarland

**Affiliations:** 1 University of California San Francisco, San Francisco, CA, USA

**Keywords:** human papillomavirus, HPV vaccine, low-income, health disparities

## Abstract

Despite widespread availability of human papillomavirus (HPV) vaccines and recommendations for routine use, awareness and uptake of HPV vaccination are not universal. We assessed self-reported history of HPV vaccination in a sample of low-income men and women recruited from the community using respondent-driven sampling as part of the National HIV Behavioral Surveillance (NHBS) survey in San Francisco. Of the 384 respondents, a minority (12.5%) reported they had received the HPV vaccine. In multivariate analysis, independent associations with HPV vaccination history were female sex (adjusted odds ratio [AOR] = 3.76, 95% confidence interval [CI] = [1.73, 8.17]), younger age (AOR = 0.89 per year, 95% CI = [0.86, 0.92]), and education above high school (AOR = 2.84, 95% CI = [1.37, 5.90]). Missed opportunities for HPV vaccination were evident in 84.4% of respondents having visited a health care provider in the last year, including 40.1% tested for a sexually transmitted infection, and entry into higher education programs (33.4%).

Human papillomavirus (HPV) is the most common sexually transmitted infection (STI) in the United States (Centers for Disease Control and Prevention [CDC], 2022b). A well-documented sequala of HPV infection is cervical cancer; however, HPV can also cause cancer of the vulva, vagina, penis, anus, and oropharynx (CDC, 2022b). Fortunately, a safe and effective vaccine to prevent the major cancer-causing serotypes of HPV has been available since 2006 (Markowitz et al., 2014). The Advisory Committee on Immunization Practices (ACIP) first recommended routine HPV vaccination for girls aged 11 to 12 years, starting as early as 9 years old and recommending catch-up vaccinations for women up to 26 years of age (Markowitz et al., 2007), later recommending vaccination for boys in 2011 and catch-up vaccinations for men up to 21 years of age (CDC, n.d.-a). Currently, there is no recommendation for adults aged 27 to 45 years, but ACIP recommends these individuals discuss with their doctors and weigh the benefits from the vaccine against their potential risk of new HPV infection (Meites et al., 2019).

Despite widespread availability and ACIP recommendations for routine use, awareness and uptake of HPV vaccination are not universal. One study found that awareness of the HPV vaccine had declined by 6.2% among females and 8% among males within the general U.S. population following the first 10 years after its introduction (Chido-Amajuoyi et al., 2021). It is well worth tracking community levels of HPV vaccination awareness and uptake, particularly among populations experiencing health disparities such as those in low-income areas. We explore HPV vaccination status among low-income, sexually active men and women in San Francisco who participated in the National HIV Behavioral Surveillance (NHBS) survey.

## Method

We conducted a secondary analysis using data from the NHBS survey collected in 2019 among low-income heterosexual men and women. The CDC leads the NHBS series of cross-sectional surveys that are conducted in 23 U.S. metropolitan areas with high HIV burden. The data used in this analysis originated from the San Francisco site only. Details of the general methods are available in the CDC’s reports (CDC, n.d.-b) and in a previous publication on awareness and use of pre-exposure prophylaxis for HIV from San Francisco (Namara et al., 2021). In brief, NHBS collects data regarding behavioral risk factors for HIV and STI, including awareness and history of HPV vaccination, in face-to-face interviews. Recruitment for the heterosexual cycle of NHBS is by respondent-driven sampling (RDS). RDS uses long chains of peer referrals to recruit eligible participants. Recruitment begins with selected “seeds” (i.e., a small number of eligible persons from different social networks) identified by health programs or through outreach. After seeds are enrolled in the survey, they are asked to refer up to five peers from their social networks. Compensation of US$50 is given for completing the survey and US$20 for each successful referral. Eligibility criteria for NHBS (CDC, n.d.-b) and the current analysis were age ≥18 years, current residence in San Francisco or San Mateo county, a maximum annual income of US$24,000, no previous participation in NHBS during the current survey cycle, ability to complete the survey in either English or Spanish, and ability to provide informed consent. In addition to these basic NHBS eligibility requirements, participation in the 2019 NHBS cycle for high-risk heterosexuals was limited to persons who (1) were aged <60 years, (2) reported vaginal or anal sex with an opposite sex partner in the 12 months before interview, and (3) reported their gender as either male or female. Participants are only included in the 2019 NHBS if they (1) never injected any drugs other than those prescribed for them, or (2) male participants who have never had oral or anal sex with another male. Exclusion criteria were previously participating in the present round of the survey, not being referred by a current participant, and unable to provide informed consent.

We used Stata version 17 software to analyze the self-reported history of HPV vaccination among low-income heterosexual men and women in San Francisco. The question on HPV vaccination history was: “Have you ever received a vaccine against HPV, for example, GARDASIL, which prevents genital warts, anal cancer, and cervical cancer?” Those who replied “Yes” were asked, “How old were you when you received your first dose of the HPV vaccine?” As in the CDC national report and previous publication (CDC, n.d.-b), data are unweighted (i.e., the sample proportions are presented). Multivariate logistic regression analysis was used to characterize associations with a history of HPV vaccination within the sample. Variables with *p* < .10 were considered for entry into a multivariate model; those with *p* < .05 were retained in the final model.

## Results

Approximately half of the survey participants were women (51.3%) and above the age of 45 years (50.3). A majority were Black/African American (82.2%) and had a high school education or greater (83.0%) (*N* = 384, Table 1). The majority of men (65.8%) and women (57.9%) reported having more than one sexual partner in the last 12 months. Most (84.4%) had visited a health care provider in the last 12 months; however, fewer (40.1%) had been tested for an STI.

**Table 1.** Characteristics and Human Papillomavirus (HPV) Vaccination History of Respondents in a Community-Based Sample of Low-Income Women and Men, San Francisco, 2019 (*N* = 384).

Characteristics	*N*	%
Sex		
Female	197	51.3
Male	187	48.7
Age group in years		
18–24	51	13.3
25–34	70	18.2
35–44	70	18.2
45 and above	193	50.3
Hispanic ethnicity	78	20.3
Race (multiple categories permitted, *n* = 371)		
American Indian or Alaska Native	30	8.1
Asian	10	2.7
Black/African American	305	82.2
Native Hawaiian or Other Pacific Islander	14	3.8
White	52	14.0
Education attainment^a^		
Eighth grade or less	5	1.3
Some high school	60	15.7
High school graduate or GED	190	49.6
Some college, associate, or technical degree	113	29.5
Bachelor’s degree	13	3.4
Any postgraduate studies	2	0.5
Dependents		
None	185	48.2
1 or more	199	51.8
Number of female sexual partners (male respondents), last 12 months^a^	64	34.2
1	71	38.0
2–3	26	13.9
4–5	12	6.4
6–8	9	4.8
9–10	4	2.7
>11		
Number of male sexual partners (female respondents), last 12 months		
1	83	42.1
2–3	72	36.6
4–5	22	11.2
6–8	10	5.1
9–10	4	2.0
>11	6	3.1
Health care provider visit, last 12 months		
Yes	324	84.4
No	60	15.6
Sexually transmitted infection test, last 12 months		
Yes	154	40.1
No	230	59.9
Have you ever received a vaccine against HPV, for example, GARDASIL, which prevents genital warts, anal cancer, and cervical cancer?	48	12.5
Yes	336	87.5
No		
How old were you when you received your first dose of the HPV vaccine? Age group in years (*n* = 46):		
10 and under	4	8.7
11–12	7	15.2
13–15	7	15.2
16–25	19	41.3
26 and above	9	19.6

*Note*. GED = General Educational Development.

a Some categories do not add up to the total number study subjects due to missing data. One participant declined to answer these questions.

A minority of participants (12.5%) reported they had received the HPV vaccine. Among those recalling the age at which they received the first dose, a plurality (41.3%) were between 16 and 25 years old; 19.6% reported they were 26 years or older.

Table 2 shows the prevalence of reported HPV vaccination by respondent characteristics. In bivariate analyses, HPV vaccination was significantly more likely to be reported by women (18.8%), younger persons (e.g., 39.2% among those aged 18–24 years), those with dependents (16.6%), and those tested for a sexually transmitted disease (STD) in the last year (21.4%). Higher education level (i.e., some college 20.4%) showed a borderline significant (*p* = .085) association with a history of HPV vaccination. In multivariate analysis, independent associations with HPV vaccination history were female sex (adjusted odds ratio [AOR] = 3.76, 95% confidence interval [CI] = [1.73, 8.17]), younger age (AOR = 0.89 per year, 95% CI = [0.86, 0.92]), and education beyond high school (AOR = 2.84, 95% CI = [1.37, 5.90]). Age confounded the relationship between HPV vaccination and having dependents and being tested for an STI in the last year. In this sample, HPV vaccination history did not significantly vary by race.

**Table 2.** Associations With Human Papillomavirus Vaccination History Among Respondents in a Community-Based Sample of Low-Income Women and Men, San Francisco, 2019 (*N* = 384).

Characteristics	History of HPV vaccination *N* (%)	Bivariate *p* value	Adjusted odds ratio [95% confidence interval]^a^
Sex			
Female	37 (18.8)	<.001	3.76 [1.73, 8.17]
Male	11 (5.9)		
Age group in years			
18–24	20 (39.2)		
25–34	19 (27.1)	<.001	0.89 [0.86, 0.92] per year
35–44	6 (8.6)		
45 and above	3 (1.6)		
Ethnicity			
Hispanic	10 (12.8)	.924	—
Non-Hispanic	38 (12.4)		
Race (multiple categories permitted, *n* = 371)			
American Indian or Alaska Native	2 (6.7)	.286	—
Asian	2 (20.0)	.500	—
Black/African American	40 (13.1)	.827	—
Native Hawaiian or Other Pacific	3 (21.4)	.335	—
Islander			
White	6 (11.5)	.746	—
Education attainment			
Eighth grade or less	0 (0.0)		
Some high school	5 (8.3)		
High school graduate or GED	19 (10.0)		
Some college, associate, or technical degree	23 (20.4)	.085	2.84 [1.37, 5.90]^b^
Bachelor’s degree	1 (7.7)		
Any postgraduate studies	0 (0.0)		
Dependents			
None	15 (8.1)	.012	—
1 or more	33 (16.6)		
Multiple sexual partners in the last 12 months	33 (13.9)	.284	—
Yes	15 (10.2)		
No			
Health care provider visit, last 12 months			
Yes	43 (13.3)	.288	—
No	5 (8.3)		
Sexually transmitted infection test, last 12 months			
Yes	33 (21.4)	<.001	—
No	15 (6.5)		

*Note*. HPV = human papillomavirus; GED = GED = General Educational Development.

a Included in the final model if *p* < .05 in multivariate analysis.

b Compared with other educational levels.

## Discussion

Our study found that only one in eight young adults (18–24 years) living in low-income neighborhoods of San Francisco could recall having been vaccinated for HPV. The CDC reports five in 10 adolescents (13–17 years) to be up to date with their HPV vaccine (CDC, 2022a). Our data also noted a variability in HPV vaccination history by sex, age, and education level. Women were more than 3 times as likely to report HPV vaccination compared with men. This finding may echo the former guidelines for vaccinating women prior to including men, with men failing to catch up over time. Alternatively, women may engage more regularly with sexual and reproductive health care or have better recall of HPV vaccination compared with men. Findings from a study on trends and predictors for HPV vaccination among U.S. college females and males in 2013 found a similar trend, where 59.0% of females versus 29.8% of males had received at least one dose of the vaccine (Thompson et al., 2016). We also found younger people were more likely to report HPV vaccination, which may reflect newer guidelines that routinized HPV into childhood and school-aged vaccination schedules (Markowitz et al., 2014). Notably, the finding of increased HPV vaccination with increasing education level was significant after controlling for increasing age, suggesting enrollment in higher education afforded increased access to preventive care.

We recognize the limitations of our study. First, as this is a secondary analysis of data collected for the NHBS, our examination of HPV vaccination was not structurally planned and therefore has the limitations of inclusion of participants and the content of the questionnaire. Second, data included only heterosexual men and women as being eligible for the NHBS heterosexual survey round. Examination of HPV vaccination among other groups at high risk, such as men who have sex with men (MSM) and trans women, can be done with future NHBS surveys for these populations. Third, data are self-reported and not verified by medical record examination or serological testing. Fourth, NHBS eligibility criteria did not include the younger age groups recommended for vaccination. Fifth, the NHBS questionnaire did not include the number of doses received. Another limitation is the representativeness of the sample. Recruitment through peer referral may create biases that are difficult to measure and correct for. Nonetheless, a strength of our sample is that recruitment was independent of clinics and therefore included persons who may not have access to services.

Despite limitations, there are several programmatic implications based on these data. Those in low-income neighborhoods, regardless of the race, show a low HPV awareness and low HPV vaccination rate. On a positive note, a large majority of the population had visited a health care provider in the preceding 12 months, including many who were tested for STDs. Physician visits are opportunities for providing information and applying interventions. In Denver, also an urban setting, Denver Health applied several interventions to increase vaccination rates among adolescents from 2004 to 2014; these included the “bundling” of three adolescent vaccinations (Tdap, MCV4, HPV) during routine physician visits and providing training to physicians regarding offering the vaccines and addressing vaccine refusal. At the end of this period, Denver Health achieved vaccination rates that were above national coverage rates (Farmar et al., 2016). Public awareness surrounding HPV can be increased with educational campaigns focused on adolescents which include social media sponsored ads, which have been proven to increase awareness among this group (Ortiz et al., 2018) as well as adults (Lama et al., 2021). Outreach campaigns targeted to parents who might associate the HPV vaccine with an active sexual life might help bridge the gap; these, however, must be culturally humble and sensitive to each audience. HealthyPeople 2030, a report set by the Office of Disease Prevention and Health Promotion to identify public health priorities over a 10-year period, has listed as an objective to increase HPV vaccination rates to 80% among adolescence aged 13 through 15 years. Most current data on the Healthy People website show this rate at 54.5% as of 2020 (Healthy People 2030, n.d.). The above target needs to be addressed simultaneously to break down the barriers to HPV awareness and vaccination. Programs that can leverage all three—physician visits, public awareness, and outreach—stand the best chance of rapidly closing the gap in HPV vaccination.
